# Cell line modeling for systems medicine in cancers (Review)

**DOI:** 10.3892/ijo.2013.2202

**Published:** 2013-12-02

**Authors:** NAYOUNG KIM, NINGNING HE, SUKJOON YOON

**Affiliations:** Center for Advanced Bioinformatics and Systems Medicine, Department of Biological Sciences, Sookmyung Women’s University, Seoul 140-742, Republic of Korea

**Keywords:** cancer cell line modeling, systems medicine, NCI60, drug sensitivity

## Abstract

Unexpected drug efficacy or resistance is poorly understood in cancers because of the lack of systematic analyses of drug response profiles in cancer tissues of various genotypic backgrounds. The recent development of high-throughput technologies has allowed massive screening of chemicals and drugs against panels of heterogeneous cancer cell lines. In parallel, multi-level omics datasets, including genome-wide genetic alterations, gene expression and protein regulation, have been generated from diverse sets of cancer cell lines, thus providing a surrogate system, known as cancer cell line modeling, that can represent cancer diversity. Taken together, recent efforts with cancer cell line modeling have enabled a systematic understanding of the causal factors of varied drug responses in cancers. These large-scale association studies could potentially predict and optimize target windows for drug treatment in cancer patients. The present review provides an overview of the major types of cell line-based large datasets and their applications in cancer studies. Moreover, this review discusses recent integrated approaches that use multi-level datasets to discover synergistic drug combination or repositioning for cancer treatment.

## Contents

1. Introduction
2. Large-scale datasets from cell line panels
3. Systematic analysis of multi-level omics and chemical screening data
4. Perspectives

## 1. Introduction

Cancer cells exhibit varied responses to anticancer agents (1). Fast high-throughput determinations of genome-wide genetic alteration, gene expression and protein regulation patterns in large collection of cancer cell lines are currently becoming key technologies with which to link the heterogenic properties of cancer cells to varied drug responses. The currently available large diverse cancer cell line collections are considered surrogate systems that can efficiently represent the complexities of primary tumor samples. Parallel datasets of common cell line panels have been widely created and analyzed to identify association patterns between phenotypes (e.g., drug responses) and intracellular signatures (e.g., mutations, gene expression or protein regulation) (2,3). Several statistical frameworks have been reported for cell line modeling, and these are mainly focused on fast determinations of mutational or molecular signatures to explain or predict unexpected drug responses in cancer subtypes (4,5). Recent studies have shown that cell line modeling could potentially predict *in vivo* anticancer drug responses or optimize target treatment windows in clinical trials.

The goals of this review are to survey the major types of cell line-based high-throughput datasets and highlight their applications in the systematic modeling of selective drug responses in cancer samples. This review focuses on several representative types of large datasets, including genotyping, gene and protein regulation, and chemical screening from well-defined cancer cell line panels. The major analytical efforts conducted with these representative datasets will be described, together with systematic approaches to integrate the multi-level omics and drug data. We expect that the present review will provide clear insights into the future impact of *in vitro* cell line modeling in translational cancer studies.

## 2. Large-scale datasets from cell line panels

**Figure 1.**
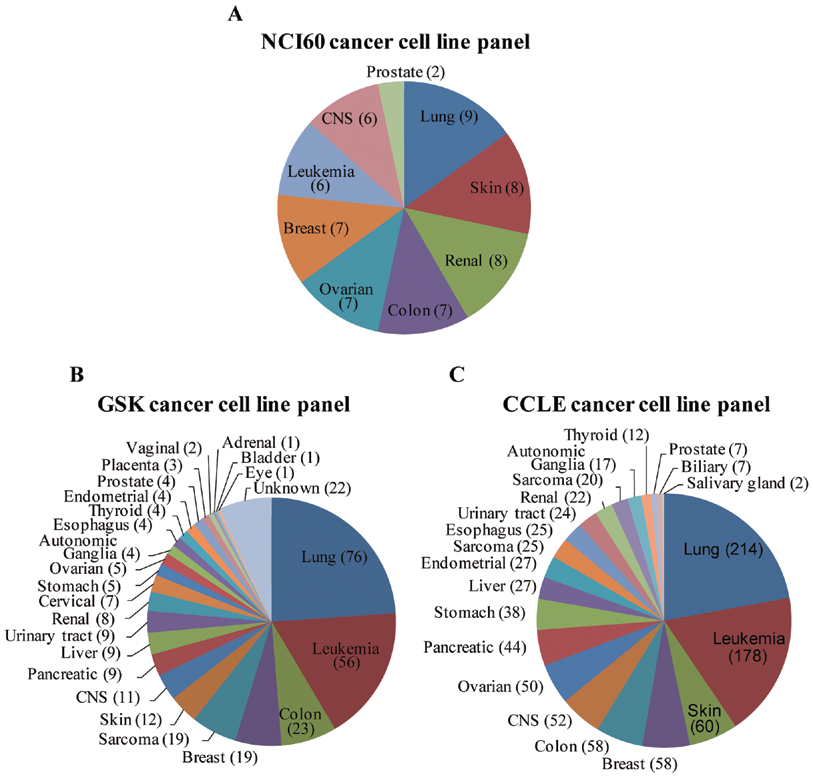
Lineage distributions of cancer cell lines in large datasets. (A) The NCI60, (B) GSK and (C) CCLE datasets include 60, 318 and 967 cell lines, respectively.

Several cancer cell line panels have been organized to perform large-scale chemical screening and multi-level omics data profiling. For example, the National Cancer Institute (NCI) developed a panel of 60 well-characterized cancer cell lines from diverse tumor types for the purpose of chemical screening against heterogeneous cancer subtypes (6). This panel, the NCI60 cancer cell line panel, includes cell lines from the 9 most frequent cancer lineage types (Fig. 1A). This panel has long been used as a standard platform, on which >40,000 chemicals were screened over the last few decades. Recently, multiple efforts have been exerted to generate genome-wide genetic variation, transcription and translational regulation data for the NCI60 cell lines. Together with these newly created omics data, the large amount of accumulated chemical screening data from the NCI60 panel are recognized as valuable resources with which to understand varied chemical responses and their underlying mechanisms.

More recently, the sizes of cell line panels for chemical screening and omics data generation have greatly increased. For example, GlaxoSmithKline (GSK) released various genomic profiling datasets from a panel of >300 cancer cell lines that comprised 24 different cancer lineages (Fig. 1B) (7). In particular, cell lines from lung and leukemia cancers comprised 42% of the panel. In addition to omics profile data, many important cancer drugs and drug candidates have been screened against this panel. The extended size of this cell line panel enables further analyses of drug responses and cancer signature regulation with regard to cancer subtypes and detailed genotypes. Another large dataset, The Cancer Cell Line Encyclopedia (CCLE) is a compilation of genomic profiling and chemical screening data launched by Novartis and the Broad Institute (3). This collection of nearly 1,000 cancer cell lines encompasses 21 cancer types and thus includes most of the well-characterized cell lines available in public resources (Fig. 1C). We expect that the GSK and CCLE datasets will synergize with the traditional NCI60 datasets with respect to emerging trends in cancer cell line modeling to facilitate an understanding and predictions of cancer progression and drug responses. Details of the current efforts conducted with these three large datasets and other cell line resources will be described and discussed below.

### Genotype profiling

Genotypic variation among cancer cells is the major cause of inconsistency in anticancer drug responses. The prospect of targeted cancer therapies relies mainly on extensive information on the genetic variations observed in diverse cancer types. Recent efforts based on high-throughput PCR and sequencing technologies have generated reliable annotations of genome-wide genetic alterations in large cancer cell line and tissue sample collections (8,9). For example, the COSMIC (Catalogue of Somatic Mutations in Cancer) Sanger database was designed to provide information on somatic mutations in human cancers (10). All the cancer mutation data were manually curated from the scientific literature, together with experimental data from the Cancer Genome Project at the Sanger Institute. The recent version of the COSMIC database (version 66), released in July, 2013, describes >1,524,000 coding mutations in approximately 909,000 cancer samples and contains both patient tumor samples and most well-known cancer cell lines (Table I). This database provides well-organized information regarding the established and annotated somatic mutations as well as previously unknown genetic alterations in potentially oncogenic factors. The cell line genotypes included in screening panels such as the GSK and CCLE datasets were, in practice, retrieved from COSMIC database.

In addition to somatic mutations in coding regions, SNPs and copy number alterations in cancer cells have been proven to be of significant importance for understanding of cancer progression and therapies. For example, cancer subtype-specific SNP markers or CNA exhibited powerful predictions regarding drug responses, clinical prognostics and oncogenic factor identification (11,12). In 2005, whole genome-based SNP and copy number alteration data were generated from the NCI60 cell lines (13). These data determined the genotypes of >124,000 SNP alleles, which can be downloaded from the NCI DTP website (Table I). In association studies of these genetic alteration data from NCI60 cell lines, several reports identified novel cancer targets (13) and signature genes responsible for drug sensitivity or resistance (14). Detailed information regarding genome-wide genetic alteration profiles in multiple cell lines can be applied to studies of diverse tumorigenic and survival mechanisms across heterogenic cancer subtypes (15,16). Furthermore, whole-exome sequencing data were recently released for the NCI60 cell lines (Table I) (17). Together with the diverse genomic variants displayed among the tumor types, 16 cancer genes were newly discovered during this large-scale exome sequencing. Furthermore, coding variant- specific drug response profiling suggested novel hypotheses that were applicable to the identification of previously unknown pharmacogenomic correlations. This extensive genotypic information on the NCI60 panel is expected to play a critical role in interpreting the large amounts of chemical screening data from the same cell lines.

### Gene expression profiling

Gene expression analysis in cancers has provided considerable information on diagnostic or prognostic marker signatures and potential drug targets. DNA microarray experiments have generated substantial transcriptome-wide gene expression profile information in various cancer samples. A DNA microarray dataset of the NCI60 cell lines was initially generated to explore the expression of approximately 8,000 unique genes (Table II) (18). This dataset was applied to analyze gene expression patterns in cancer type classifications (19). More recently, extensive profiles of >25,000 genes were generated from the NCI60 cell lines. These data have been used to predict target gene signatures and identify unique gene signatures with respect to cancer mechanisms, regulatory pathways and functional categories (20). We expect that the gene expression data from NCI60 panel will be useful for identifying gene signatures of drug sensitivity or resistance.

Cancer transcriptome expression profiles were also generated for the GSK and CCLE cell lines. The GSK dataset includes gene expression data for >300 cell lines, each in triplicate, thus providing a robust statistical analysis (7) (Table II). This dataset enabled many different types of association studies such as those of subtype- or mutation-dependent gene expression patterns in various cancer lineages (1). These association studies revealed that the expression levels of some drug target genes were associated with previously unknown mutational status in several lineage groups. The CCLE cell line panel microarray dataset was analyzed in combination with genotype profiling and chemical screening data (3) (Table II). During this combined analysis, the AHR gene was identified as a predictor of mutation-based drug sensitivity. AHR gene expression was associated with sensitivity to a MEK inhibitor in NRAS-mutant cells. This facilitated the establishment of a preclinical cancer cell line model (3). The GSK and CCLE panels include large numbers of cell lines, thus enabling sub-classifications of cancer lineages and mutation types in statistical analyses. Clearly, transcriptome data from GSK and CCLE will be applied to a systematic understanding of cancer progression and drug responses in diverse genetic backgrounds.

### Protein expression and activation profiling

Although large-scale gene expression studies have yielded useful information for cancer biomarker identification and targeted cancer therapy development, high-throughput protein expression and activation level screening are required in order to better understand cellular signaling in the contexts of tumorigenesis and drug response. Reverse-phase protein array (RPPA) technologies, which are based on sample spot arrays for specific antibody reactions, allow fast, quantitative measurements of protein expression or phosphorylation in a large cancer sample panel (21). More than 200 general expression or phospho-antibodies for major cancer signaling molecules have been used to develop an HTS-format RPPA experiment. Several studies have shown that RPPA technology can effectively map intracellular signaling networks in various cancer sample panels, including the NCI60 (4) and GSK (1) panels (Table II). RPPA datasets that were generated from the NCI60 panel demonstrated an association between cancer subtypes and particular protein expression or phosphorylation patterns and thus provided new insights into subtype-specific signaling networks. The RPPA data revealed 5 major clusters of cell lines and 5 principal proteomic signatures. Cell lines with PTEN, PIK3CA, BRAF and APC mutations were found to be significantly associated with proteomic clusters (4). Additionally, RPPA data for >150 cell lines in the GSK panel enabled a mutation-oriented analysis of protein regulation in cancers (1). This analysis found that the major signaling network-specific signatures were well-clustered in a mutation-based cell line classification. Specifically, the analysis revealed that the MEK1, MAPK and p90RSK signatures in the MAPK/Erk signaling networks had distinct regulatory patterns in BRAF-mutant cell lines. The application of RPPA experiments to target core signaling proteins in cancers provides unprecedented opportunities for a system-level understanding of the molecular signaling mechanisms in cancer progression and drug responses. The increased diversity of appropriate antibodies for RPPA experiments will further contribute to cell line modeling technologies, together with transcriptome data.

### Chemical and drug screening - GI50 profiling

A panel of well-characterized cancer cell lines, exhibiting diverse cancer types and varied genetic alterations, have provided a platform for chemical screening and prediction of cancer subtype-specific drug responses. A large number of anti-cancer agents have been screened on well-defined sets of cancer cell lines (Table II). The NCI60 project was the first large-scale and systematic approach to anticancer chemical screening (22). This project has accumulated GI50 profiles for >50,000 compounds screened against a panel of 60 cell lines. These screening data, together with chemical structures, are freely available through the project website (http://dtp.nci.nih.gov/index.html). This is the largest dataset for studies of structure-activity relationships between anticancer agents and diverse cancer subtypes. The COMPARE program is a useful tool with which to search for compounds with similar patterns of cellular sensitivity in the NCI60 panel (23). By extensively using the COMPARE program, it is possible to compare the expression (or activation) patterns of a gene (or protein) to GI50 data from 60 cell lines (24).

More extensive sets of cell lines, compared to NCI60, were also used to screen major classes of cancer drugs and developmental candidates. The dataset provided by McDermott *et al* (25) included the GI50 profiles of 14 selective kinase inhibitors against 500 human cancer cell lines (Table II). This dataset provided new hypotheses for discoveries of sensitive genotypes for a given kinase inhibitor, in addition to lineage-dependent compound responses. The GSK dataset provided screening data for 19 target-defined compounds against 311 diverse human cancer cell lines (7) (Table II). An analysis of this dataset showed that major cancer genes played critical roles in the dose-dependent inhibition of cell proliferation by selective kinase inhibitors (26). An integrated analysis of these two large datasets distinguished the major cancer lineages and genotypes that were selectively sensitive to given kinase inhibitors (1). Another large-scale drug screening dataset includes 130 anticancer agents screened against 639 cell lines (Table II). This dataset was used to identify and develop sensitive cancer therapeutic biomarkers (27). The CCLE panel was also used to build a model that showed single and complex gene-drug associations in order to explain the range of drug sensitivities across cell lines (3). This model included screening data for 24 anticancer agents across 479 cancer cell lines (Table II).

Additionally, *in silico* molecular modeling technology, coupled with the increased availability of cell line screening profiles, has created new opportunities for novel selective cancer drug design. For example, systematic analyses, using 2D and/or 3D-structure chemical descriptors, attempted to accurately classify compounds to predict their varied responses against diverse cancer cell lines (28). Cell line panels that are annotated with both genetic alterations and compound screening data provide an unprecedented surrogate tool for the prediction of compound sensitivity specific to narrow cancer subtype ranges. The generation of chemical screening data against large numbers of cell lines has provided robust statistical analysis for systematic analyses of the relationships between drug responses and cancer genetic markers.

## 3. Systematic analysis of multi-level omics and chemical screening data

The availability of diverse datasets from cell line panels presents new approaches to predictions of drug sensitivity or resistance that are based on the genetic alteration status, lineage types and gene or protein markers of the cell lines. Many attempts have been made to use integrated system-level analyses of omics and drug response datasets from cell line panels. The NCI60 cell line panel has accumulated large amounts of chemical screening data, together with multi-level omics data that include genome-wide genetic alterations, gene expression and protein regulation. Thus, the NCI60 datasets have been widely used for integrated systematic analyses of drug responses and cancer progression mechanisms. Typical association studies with linear correlation patterns attempted to identify relationships among chemical responses, genetic alterations, mRNA expression and protein regulation in 60 cell lines (2). Compared to the chemical response data, observed pattern similarities in omics datasets can be interpreted as underlying molecular cellular response mechanisms to the chemicals.

The genotypic variations in cancer cell lines provide a basis on which to understand variability in chemical responses and predictive biomarker identification (29). Integrated proteomic data analyses revealed protein expression or activation signatures that could explain mutation-specific chemical sensitivity among heterogeneous cancer cell lines (4). A ‘Connectivity Map’ has been created to quantitatively explain the connections between genome-wide gene expression patterns and drug responses in a cell line (5). Another statistical framework, the cell line enrichment analysis (CLEA), was introduced to integrate multi-level omics datasets and chemical screening data from a cell line panel (1). In cases such as the GSK or CCLE datasets, which included a large number of cell lines, it was possible to generate significant statistical confidence in association studies between the datasets by using refined cell line classification categories such as multiple mutation types or mutation-lineage combinations. The CLEA study presented many gene or protein signatures associated with specific chemical responses in the double mutation or mutation-lineage combination categories (1). The Cancer Genome Project (CGP) (27) and CCLE (3) datasets include chemical screening data against larger cancer cell line panels than NCI60 (Table II). The CGP revealed unexpected relationships, including the marked sensitivity of Ewing’s sarcoma cells that harbor EWS-FLI1 gene translocation to PARP inhibitors. In the case of CCLE, incorporated analyses of drug responses, gene expression, copy number sequencing and genomic cell line characterizations were used to identify several novel drug sensitivity predictors. This system-level integration of cell line datasets provides new tools for understanding the diversity of cancer progression and to develop synergistic drug combinations for target cancer subtypes.

## 4. Perspectives

The availability of large-scale, multi-level omics and chemical screening datasets for well-characterized cell lines will accelerate system-level studies of cancer progression and the development of improved therapeutics. In particular, the recent explosion of genome-wide exome sequencing and RNA sequencing data further contributes to the refined characterization of diverse cancer cell lines and better interpretation of chemical screening data obtained using these lines. The Cancer Genome Atlas (TCGA) is another exciting project that has extended ideas about cell line modeling to human cancer tissue samples. An increasingly large collection of clinical cancer samples are directly used to generate multi-level omics data, thus revealing a comprehensive landscape of genetic alterations and transcriptional regulation in each cancer subtype (30). A recent study identified *de novo* sets of genes through a correlation analysis of gene expression profiles from the NCI60 and TCGA datasets (31). Together with patient history information, including drug treatments and metastases, TCGA datasets have synergistically contributed to cell line modeling applications for the prediction of drug responses and molecular signatures in cancers.

Together with systematic cell line modeling using large omics datasets, RNAi screening data were recently generated to identify the target genes associated with changes in cancer phenotypes. For example, a kinome-based shRNA screening study was performed to determine the mechanism of resistance against BRAF inhibitors in colon cancers that harbored an activating mutation in the BRAF oncogene (32). Feedback EGFR activation, along with BRAF inhibition, was identified as the key factor behind the resistance. Another shRNA screening study identified target genes that effectively cooperated with MEK inhibitors in KRAS-mutant cancers, suggesting a therapeutic potential for a combination of MEK and BCL-XL inhibitors (33). Integrative analyses of siRNA screening and proteomic RPPA data from the NCI60 cell lines revealed diverse kinase signaling network connectivity (34). This approach revealed a novel interaction between GSK3 and AKT phosphorylation in cancers.

## Figures and Tables

**Table I.** Databases of cancer sample genotype profiles.

Database	Data size	Description
COSMIC (http://www.sanger.ac.uk/cosmic)	909,000 cancer samples 904,000 tissues 5,000 cell lines	Collection of 1,524,000 coding mutations (version 66, July 2013) -Manually curated from scientific literature -Sequence variants/mutations from the Cancer Genome Project at Sanger Institute
NCI60	60 selected cell lines	>124,000 SNP alleles (Affymetrix 125K SNP array)
(http://dtp.nci.nih.gov/mtargets/download.html)		All variations on 38 Mb of coding regions -Exome sequencing (Agilent SureSelect All Exon v1.0)

**Table II.** Representative cell line-based datasets with large gene expression, protein regulation and chemical screening data profiles.

Cell line panel	Data type	Description
NCI60	Gene expression (DNA microarray)	Expression profiles of 9703 genes in 60 cell lines (NCI cDNA array) Expression profiles of 54,613 gene probes in 60 cell lines (Affymetrix U133 version 2)
	Protein expression and phosphorylation (RPPA experiment)	NCI DTP dataset (RPPA experiment) -Profiles of 89 proteins in 60 cell lines (68 expression and 21 phospho-antibodies) MDA_class dataset -Profiles of 99 proteins in 60 cell lines (65 expression and 33 phospho-antibodies). MDA_pilot dataset -Profiles of 34 proteins in 60 cell lines (25 expression and 9 phospho-antibodies)
	Chemical screening	GI50 of >50,000 chemicals in 60 cell lines
CCLE^a^	Gene expression (DNA microarray)	Expression profile of 54,613 gene probes on 967 cell lines (Affymetrix U133 v2)
	Chemical screening	GI50 of 24 chemicals on 479 cell lines
GSK^a^	Gene expression (DNA microarray)	Expression profiles of 54,613 gene probes in 318 cell lines^b^ (Affymetrix U133 version 2)
	Protein expression and phosphorylation (RPPA experiment)	Profiles of 115 proteins in 170 cell lines (77 expression and 38 phospho-antibodies)
	Chemical screening	GI50 of 19 drugs and drug candidates in 311 cell lines GI50 of 14 kinase inhibitors in 500 cell lines
CGP^a^	Chemical screening	GI50 of 130 chemicals in 639 cell lines

a CCLE, Cancer Cell Line Encyclopedia; GSK, Glaxo Smith Kline; CGP, Cancer Genome Project.

b A total of 950 arrays performed in triplicate for each cell line.
